# Identification of CD101 in Glioma: A Novel Prognostic Indicator Expressed on M2 Macrophages

**DOI:** 10.3389/fimmu.2022.845223

**Published:** 2022-03-08

**Authors:** Yuyang Liu, Renqi Yao, Ying Shi, Yuxiao Liu, Hongyu Liu, Jialin Liu, Yunqian Guan, Yongming Yao, Ling Chen

**Affiliations:** ^1^Medical School of Chinese People’s Liberation Army (PLA), Beijing, China; ^2^Senior Department of Neurosurgery, the First Medical Center of People’s Liberation Army (PLA) General Hospital, Beijing, China; ^3^Translational Medicine Research Center, Medical Innovation Research Division and Fourth Medical Center of the Chinese People’s Liberation Army (PLA) General Hospital, Beijing, China; ^4^Department of Burn Surgery, the First Affiliated Hospital of Naval Medical University, Shanghai, China; ^5^School of Medicine, University of Electronic Science and Technology of China, Chengdu, China; ^6^Cell Therapy Center, Xuanwu Hospital, Capital Medical University, Beijing, China

**Keywords:** glioma, macrophage, CD101, immune infiltration, prognostic biomarker

## Abstract

Glioma represents the most common primary intracranial malignancy worldwide, with low overall survival rates and limited therapeutic options. The protein CD101, mainly expressed on several immune cells, has been demonstrated to exert potent effects on blunting T cell immune responses across infectious and autoimmunity diseases. Nevertheless, the prognostic value of CD101 expression and its role in the immune microenvironment of various malignancies currently remains elusive. Herein, by adopting bioinformatics methodology, we comprehensively illustrated the potential function and predictive value of CD101 in stratifying clinical prognosis among patients with glioma, for which a high CD101 level predicted an unfavorable clinical outcome in glioma patients. Results from enrichment analyses manifested that CD101 predominantly expressed on the tumor-associated macrophages and was significantly associated with the immune regulatory processes, as evidenced by its positive correlation with immune-related genes and the putative infiltration of immune cells. Evidence provided by *in-situ* multicolor immunofluorescence staining further validated our findings at the protein level. Taken together, CD101 may serve as a novel biomarker in predicting clinical prognosis and immune status for glioma patients.

## Introduction

Glioma represents the common type of primary intracranial malignancy yet accounts for the leading cause of brain cancer-related deaths. Among adult individuals, glioma can be further categorized into II to IV grades based on WHO recommendation (1). Although multimodal regimens have been introduced so far, including surgical resection, chemotherapy, radiotherapy, and immune-adjuvant therapy, the prognosis of patients with glioma remained unsatisfactory (2). Glioblastoma, a grade 4 glioma, is deemed as the most fatal form associated with blunted treatment efficacy, for which the 2-year survival rate is merely 26.5%, with a median survival duration of 15 months (3). Meanwhile, there still exist several low-grade gliomas (LGG) that maintain a low response rate to routine treatment (4). Recent studies have revealed that the tumor microenvironment (TME) is the key player in facilitating malignant growth and immune evasion (5). To be specific, the extracellular matrix (ECM), parenchyma cells, soluble factors, and infiltrating immune cells are essential components in constituting the TME of glioma (6), in which various subsets of tumor-infiltered myeloid cells played an indispensable role in responses to immunotherapies, cancer-induced immunosuppression, and tumor recurrence, especially for the presence of tumor-associated macrophages (TAMs) (7). Therefore, the identification of specific yet robust immune-relevant biomarkers reflecting the functional status of TAMs in glioma is of prominent significance.

Previous studies have demonstrated that the CD101 gene encodes a transmembrane glycoprotein predominantly expressed on dendritic cells, monocytes, and T cells (8). Of note, recent work of CD101 mainly focused on its role in restraining T cells in inflammatory processes including infectious (9, 10) and autoimmunity diseases (11, 12). CD101 was demonstrated to exert a potent effect on dampening T cell proliferation and activation in a TCR/CD3-dependent manner, as supported by the suppressed expression of IL-2RA and diminished secretion of IL-2 (8, 13, 14). The immunoregulatory potential of CD101 was further strengthened by a subsequent study involving graft versus host disease, in which they manifested that an elevated expression level of CD101 on Tregs was associated with an increased capacity in restraining effector T cells. Recently, CD101 was characterized as one of the hallmarks of T cell anergy (15). Likewise, CD101 variants can also alter the function of T cells by mediating Treg cell dysfunction and increasing T cell activation, thereby contributing to the homeostatic regulation of inflammation (10). Nevertheless, as a molecule expressed on diverse immune cell populations, the role of CD101 in many other cell types and human diseases remains largely unknown.

To the best of our knowledge, the current study is the very first report investigating the predictive value of CD101 in glioma patients. Our work revealed that CD101 could serve as an independent prognostic indicator, the upregulation of which is positively correlated with unfavorable overall survival (OS) among glioma patients. Enrichment analysis implicated that ECM, immune effector process, immune receptor activity, and humoral immune response were associated with upregulated CD101 expression. Moreover, analysis of CD101-interacting molecules reflected that CD101 might have an intimate relationship with the isogenic ligand expressed on the T cells. Additionally, immune infiltration analysis uncovered the association of CD101 with immunosuppressive status in TME. Finally, based on the bioinformatics analysis on cell type-specific expression and experimental validations using clinical specimens, M2-like TAMs were found to uniquely express a high level of CD101. These data shed light on the cellular and molecular basis of the glioma immune microenvironment, thereby guiding the development of immunomodulatory strategies in glioma.

## Materials and Methods

### Dataset Collection and Normalization

The RNA-seq data for normal brain tissues were downloaded from the GTEx database (16). Clinical information and corresponding gene expression data of 695 samples (TCGA) were obtained from the UCSC Xena database (https://xena.ucsc.edu/). The raw data were normalized with the transcripts per million (TPM) method, and log_2_ (TPM+1) transformation was applied for the subsequent analyses.

### CD101 Expression Analysis

R software (Version 3.6.3) was used for statistical analysis. The “ggplot2” package was adopted for visualization. The GEPIA2021 database (17) (http://gepia2021.cancer-pku.cn/) was implemented to analyze the immune cell type-specific expression of CD101 and to infer the immune cell composition in glioma. Moreover, the representative immunohistochemistry (IHC) staining and single-cell expression level of CD101 were retrieved from the Human Protein Atlas (HPA) online database (http://www.proteinatlas.org). The table box plots were used to present the CD101 expression level of patients stratifying by different characteristics including WHO grade, integrated diagnosis, age, isocitrate dehydrogenase (IDH) status, 1p/19q codeletion, and primary therapy outcomes.

### Differentially Expressed Gene Analysis

Differentially expressed genes (DEGs) were identified between differently expressed CD101 groups (high-expression group: 50%–100%; low-expression group: 0%–50%). The “DEseq2” package was applied to perform statistical analysis. Upregulated and downregulated DEGs with an adjusted p value < 0.05 and absolute log2 fold change (FC) > 1 were processed into subsequent analysis, for which the volcano plot was used for visualization. Thereafter, the heat map was used to depict the top 10 upregulated and downregulated DEGs. Additionally, enrichment analysis was adopted using the Metascape (https://metascape.org/) online database (18). Correspondingly, the top 20 enriched terms of the Gene Ontology (GO) and Kyoto Encyclopedia of Genes and Genomes (KEGG) were presented.

### Gene Set Enrichment Analysis

Gene set enrichment analysis (GSEA) was performed using the “clusterProfiler” package with 1,000 permutations and weighted enrichment statistics. Genes with false discovery rate (FDR) < 0.25 and p. adjust < 0.05 were of statistical significance, and the “ggplot2” package was used for visualization.

### Identification of CD101-Interacting Molecules and Functional Enrichment

A CD101-related gene–gene interaction network was constructed using the GeneMANIA database (19) (http://www.genemania.org). The CD101-associated protein–protein interaction (PPI) network was constructed using the STRING online database (20) (https://string-db.org/) and the Cytoscape software (21) was utilized for visualization. The KEGG and GO enrichment analyses were applied for analyzing CD101-binding proteins. The “clusterProfiler” package was applied for statistical analysis, and the “ggplot2” package was used for visualization.

### Glioma Immune Microenvironment Analysis

The immune score, stromal score, and estimate score were quantified by applying the “Estimate” R package. CIBERSORT (22) (https://cibersort.stanford.edu/) was utilized to measure the relative proportion of 22 human immune cell types. The correlation between the CIBERSORT score and CD101 expression was used to detect immune cell types that were possibly altered by CD101 expression. Additionally, a correlation analysis between CD101 and immune-relevant genes was implemented to further map the landscape of the CD101-related immune microenvironment. Immune-related genes were collected from Thorsson et al. (23). Moreover, the correlation between immune cell infiltration and overall survival was analyzed by the GEPIA2021 database (17).

### Survival Analysis

Kaplan–Meier survival analysis was used to determine the association of the CD101 expression level with OS in glioma patients. The glioma cohort was categorized into two groups by median CD101 mRNA expression (high-expression group: 50%–100%; low-expression group: 0%–50%). Additionally, we further performed subgroup and sensitivity analyses on OS, stratifying glioma patients by disparate clinical features. The log-rank test was applied to verify the statistical differences between the two groups. The “survival” package was applied for statistical analysis, and the “survminer” package was used for visualization.

### Predictive Efficacy of CD101

The “timeROC” package was used to perform the time-dependent receiver operating characteristic curve (ROC) analysis to illustrate the efficacy of CD101 expression in predicting 1-, 2-, and 3-year OS. The “ggplot2” package was applied for visualization.

### Univariate and Multivariate Cox Regression Analyses

To determine whether the high CD101 expression was independently associated with increased risk of mortality among glioma patients, Cox proportional hazard regression analyses were performed on TCGA database. Univariate Cox regression analyses were conducted initially, in which potentially confounding features were chosen with p < 0.1. Multivariable Cox regression analysis was subsequently carried out to confirm the independent association of CD101 expression with OS confounding for other variables. A two-sided p value of less than 0.05 was regarded as statistical significance.

### Glioma Sample Collection

This research was approved by the Institutional Research Ethics Committee of the PLA General Hospital (batch number: S2018-089-01). A signed informed consent was obtained for all participants. Fourteen paraffin-embedded glioma tissues (2 cases were grade 2, 4 cases were grade 3, and 8 cases were grade 4) were used for immunofluorescence staining. Clinical information of glioma samples are found in **Supplementary Table 1**.

### Immunofluorescence Staining

To estimate the density of the expression level of CD101 on M2-type tumor-associated macrophages, immunofluorescence assay was exploited in our research. Formalin-fixed tissues were paraffin embedded and sliced into 4-μm sections. These sections were installed on slides and managed as previously described (24). Subsequently, the goat serum containing 0.3% Triton were used for blocking brain slices at room temperature (RT). The primary antibodies, including anti-human CD101 (1:200, 26047-1-AP, Proteintech, Wuhan, China) and anti-human CD163 (1:200, CL594-16646, Proteintech) were used to incubate with slices overnight at 4°C. After being laved in PBS for three times, the slices were incubated with the secondary antibody (1:200, SA00003-2, Proteintech) for 1 h at RT, followed by staining with DAPI (MBD0015, Sigma-Aldrich). Colocation analysis and double-stained cell counts were performed by ImageJ software.

### Statistical Analysis

For bioinformatics analysis, the Wilcoxon rank-sum test was utilized to detect the statistical significance between two groups, and the comparison of multigroups was analyzed using the Kruskal–Wallis test and Dunn’s tests. The correlation between CD101 expression and other immune-relevant genes was calculated and evaluated by Spearman’s correlation coefficient. The Student *t* test was used to detect the difference in double-strained cell counts between disparate grades of gliomas. All statistical analysis was performed using R software (version 3.6.3), and two-tailed *p* < 0.05 was considered as of statistical significance.

## Results

### Elevated CD101 Expression in Glioma

**Figure 1:**
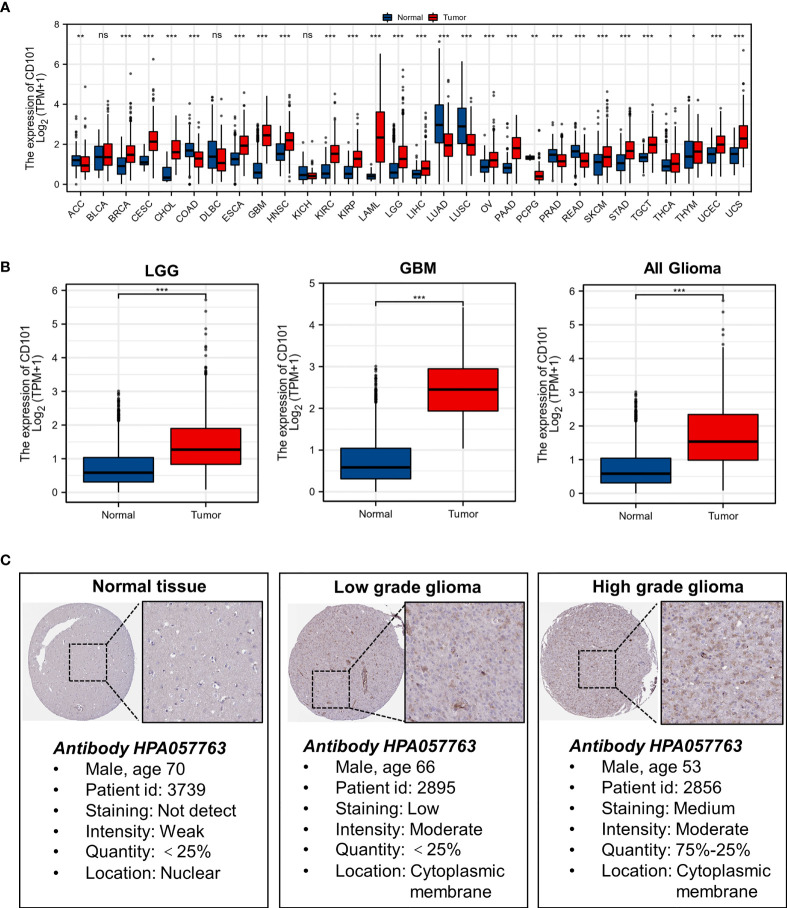
Expression level of gene CD101 in tumors and normal tissues. **(A)** CD101 expression in TCGA tumors and normal tissues with the GTEx database as controls. **(B)** CD101 expression in TCGA gliomas and normal tissues with the GTEx database as controls. **(C)** Expression of the CD101 protein was visualized by immunohistochemistry *via* the HPA database (ns, *p* ≥ 0.05, *p < 0.05, **p < 0.01, ***p < 0.001).

Results of the TCGA pan-cancer analysis revealed that a different expression level of CD101 could be observed in neoplastic sites compared to that of the normal tissues across majority of cancer types, with the exception of bladder urothelial carcinoma (BLCA), lymphoid neoplasm diffuse large B-cell lymphoma (DLBC), and kidney chromophobe (KICH) (**Figure 1A**). Specifically, we identified a significantly elevated transcript level of CD101 in low-grade glioma (LGG), glioblastoma (GBM), and all gliomas in comparison with that of the normal brain tissues (**Figure 1B**). Moreover, the *in-situ* expression of CD101 was further analyzed using HPA databases based on IHC staining, in which CD101 expression remained the highest in high-grade glioma, followed by low-grade glioma and normal brain tissue, consistent with the results from transcriptional analyses (**Figure 1C**).

### CD101 Upregulation Is Associated With Malignant Phenotypes of Glioma

**Figure 2:**
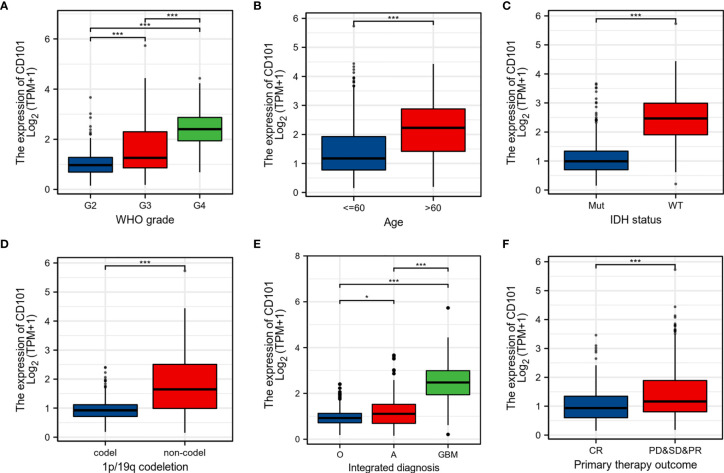
Associations between CD101 expression and different clinical characteristics in glioma. **(A)** Histological grade. **(B)** Age. **(C)** IDH mutation status, Mut: IDH-mutant; WT: IDH-wild type. **(D)** 1p/19q codeletion status. **(E)** Integrated diagnosis, O: oligodendroglioma, IDH-mutant, and 1p/19q-codeleted; A: astrocytoma, IDH-mutant; GBM: glioblastoma, IDH-wild type. **(F)** Primary therapy outcome (*p < 0.05, ***p < 0.001).

To further probe the expression pattern of CD101 in glioma, we performed subgroup analyses by stratifying patients with disparate clinical characteristics, including WHO grade, histological type, age, IDH status, 1p/19q codeletion, and primary therapy outcome. Regarding WHO grade, CD101 expression was highest in grade 4 glioma, followed by grade 3 and grade 2 gliomas (**Figure 2A**). Our data revealed a substantial increase in the CD101 level in patients older than 60 years (**Figure 2B**). In terms of IDH status, the CD101 level remained markedly enhanced in glioma tissues subjected to the IDH-wild type (**Figure 2C**). Additionally, the upregulation of CD101 was also noted in glioma tissues with 1p/19q non-codeletion (**Figure 2D**). As for integrated diagnosis, the CD101 level was significantly the highest in glioblastoma with the IDH-wild type, followed by astrocytoma with the IDH mutant and oligodendroglioma with the IDH mutant and 1p/19q-codeleted (**Figure 2E**). Stratifying by primary therapy outcome, the CD101 expression was dampened in patients with complete response to routine therapy (**Figure 2F**). These results suggested that a high CD101 expression might positively correlate with the malignant phenotypes of glioma, in association with marginal therapeutic efficacy and deteriorative clinical outcomes.

### Increased CD101 Expression Is Correlated With Unfavorable Prognosis

**Figure 3:**
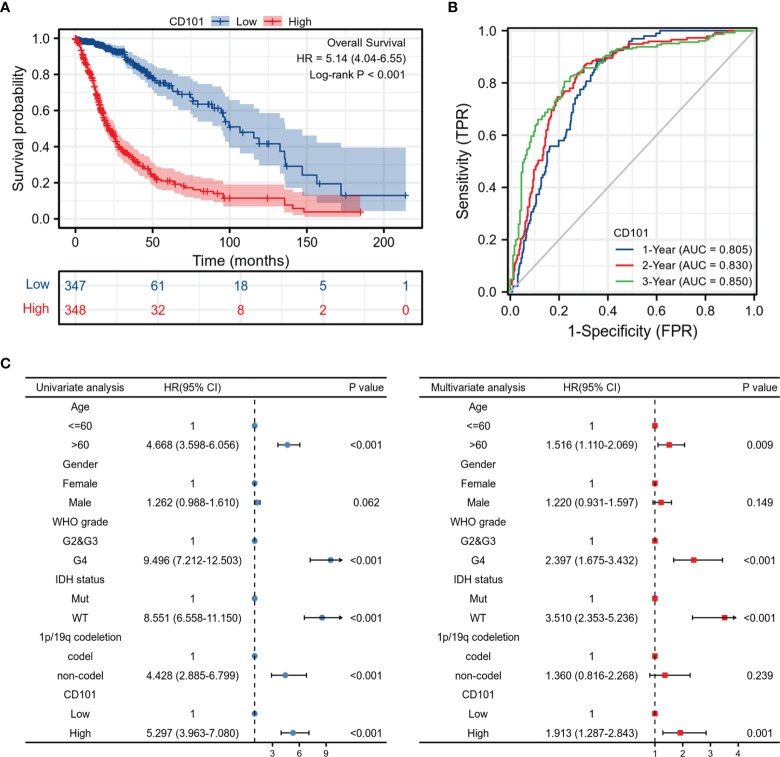
Prognostic value of the CD101 expression level in TCGA database. **(A)** Survival curves using the TCGA database are shown for OS. **(B)** Time-dependent curves for CD101 expression in glioma using TCGA database. **(C)** Forest plot of univariate and multivariate Cox regression analysis in glioma.

Since a high CD101 expression could potentially predict a malignant phenotype of glioma, we therefore examined the predictive value of CD101 in determining clinical prognosis for glioma patients derived from TCGA database (**Figure 3A**). It revealed that glioma patients with an elevated CD101 level were presented with unfavorable OS based on Kaplan–Meier survival analyses (*p* < 0.001). According to time-dependent ROC, the CD101 expression level had a relatively good performance in predicting 1-year (C statistics, 0.805), 2-year (C statistics, 0.830), and 3-year OS (C statistics, 0.850) in glioma patients (**Figure 3B**). Furthermore, univariate Cox regression analysis indicated that a high CD101 expression could potentially predict unfavorable OS (hazard ratio [HR], 5.297; 95% confidence interval [CI], 3.963–7.080; *p* < 0.001) (**Figure 3C**). After confounding for other variables, multivariate Cox regression analysis demonstrated that a high CD101 level was independently associated with increased risk of death among glioma patients (HR, 1.913; 95% CI, 1.287–2.843; *p* < 0.001) (**Figure 3C**). Taken together, a high CD101 expression level was correlated with worsening prognosis in glioma patients.

### Predictive Value of the CD101 Level Based on Clinical Subgroups

**Figure 4:**
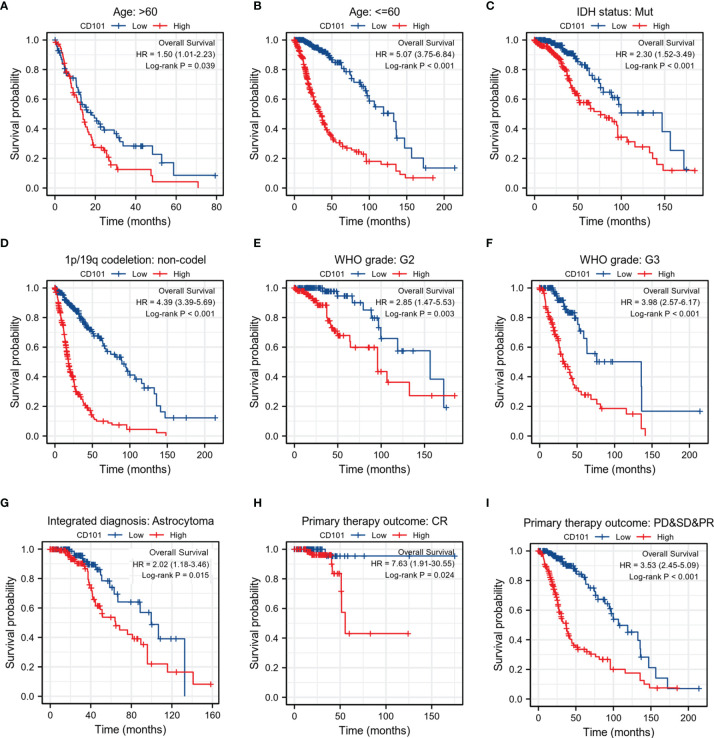
Associations between CD101 expression level and the OS in different clinical subgroups of glioma in TCGA database. **(A)** Age > 60. **(B)** Age ≤ 60. **(C)** IDH status: Mut. **(D)** 1p/19q codeletion: non-codeletion. **(E)** WHO grade: G2. **(F)** WHO grade: G3. **(G)** Integrated diagnosis: astrocytoma. **(H)** Primary therapy outcome: CR. **(I)** Primary therapy outcome: PD&SD&PR.

To validate the robustness of our findings, we subsequently investigated the correlations between CD101 expression and OS across different subgroups stratifying patients by various clinical features. The results consistently showed that glioma patients with a higher CD101 expression had a significantly deteriorative OS compared to those with a low CD101 level, including the subgroup of age >60 (**Figure 4A**), subgroup of age ≤60 (**Figure 4B**), subgroup of IDH mutation (**Figure 4C**), subgroup of 1p/19q non-codeletion (**Figure 4D**), subgroup of WHO grade 2 (**Figure 4E**), subgroup of WHO grade 3 (**Figure 4F**), subgroup of astrocytoma (**Figure 4G**), subgroup of CR (**Figure 4H**), and subgroup of PD&SD&PR (**Figure 4I**).

### Functional Enrichment Analysis of DEGs

**Figure 5:**
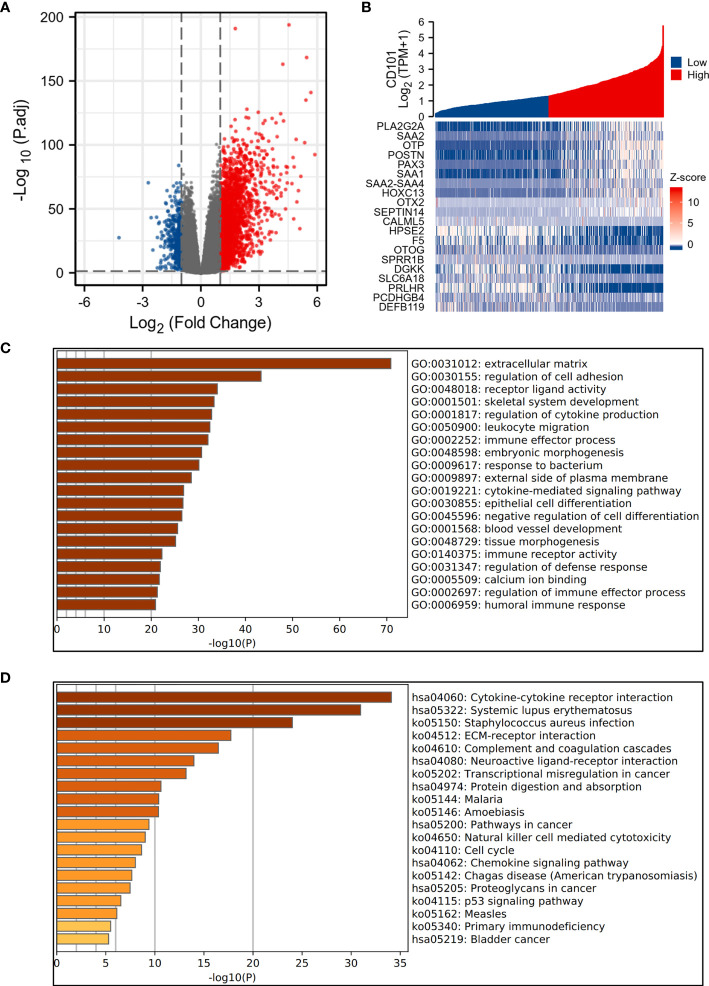
Functional enrichment analysis of 2,469 DEGs. **(A)** The volcano plot of 2,469 DEGs. **(B)** Heat maps showing the top 10 upregulated and downregulated DEGs. **(C)** Top 20 terms of GO enrichment analysis. **(D)** Top 20 terms of KEGG enrichment analysis.

**Figure 6:**
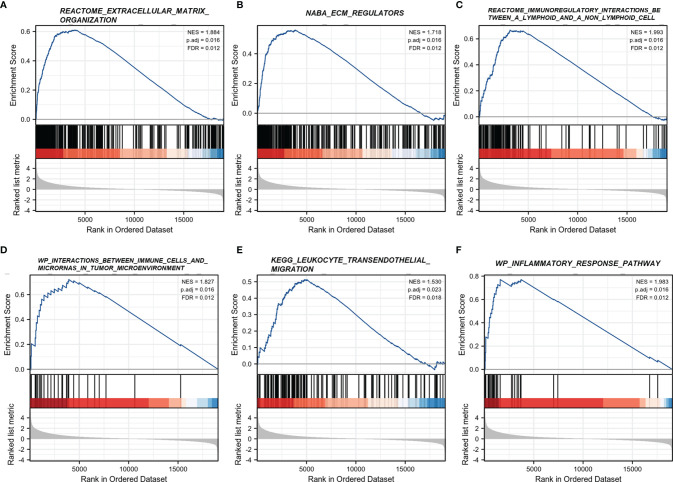
GSEA regarding the CD101 expression level. **(A)** REACTOME extracellular matrix organization. **(B)** NABA ECM regulators. **(C)** REACTOME immunoregulatory interactions between a lymphoid and a non-lymphoid cell. **(D)** WP interactions between immune cells and microRNAs in the tumor microenvironment. **(E)** KEGG leukocyte transendothelial migration. **(F)** WP inflammatory response pathway.

To interrogate the underlying effect of CD101 in glioma, we carried out functional enrichment analyses based on DEGs between patients with a high or low expression level of CD101, in which a total of 2,469 DEGs were identified accordingly, with 2,052 upregulated and 417 downregulated genes (**Figures 5A, B**). In GO enrichment analysis, the DEGs were enriched in items such as ECM, leukocyte migration, immune effector process, regulation of cytokine production, immune receptor activity, and regulation of immune effector process (**Figure 5C**). Moreover, KEGG analysis suggested that cytokine–cytokine receptor interaction, ECM–receptor interaction, transcriptional misregulation in cancer, pathways in cancer, chemokine signaling pathway, and primary immunodeficiency were potential pathways in regulating CD101 expression (**Figure 5D**). Besides, GSEA was also implemented to identify possible biological functions manipulating CD101 upregulation. Correspondingly, enrichment analysis manifested that upregulated CD101 expression was associated with extracellular matrix organization, immuno-regulatory interactions between a lymphoid and a non-lymphoid cell, and interactions between immune cells and microRNAs in the tumor microenvironment, in parallel with the results of GO and KEGG analyses (**Figures 6A–F**). These data highlighted the latent functions of CD101 in tumor immunity and ECM remodeling, rendering us to revisit its biological role in the subsequent analyses.

### Analysis of CD101-Interacting Genes and Proteins

**Figure 7:**
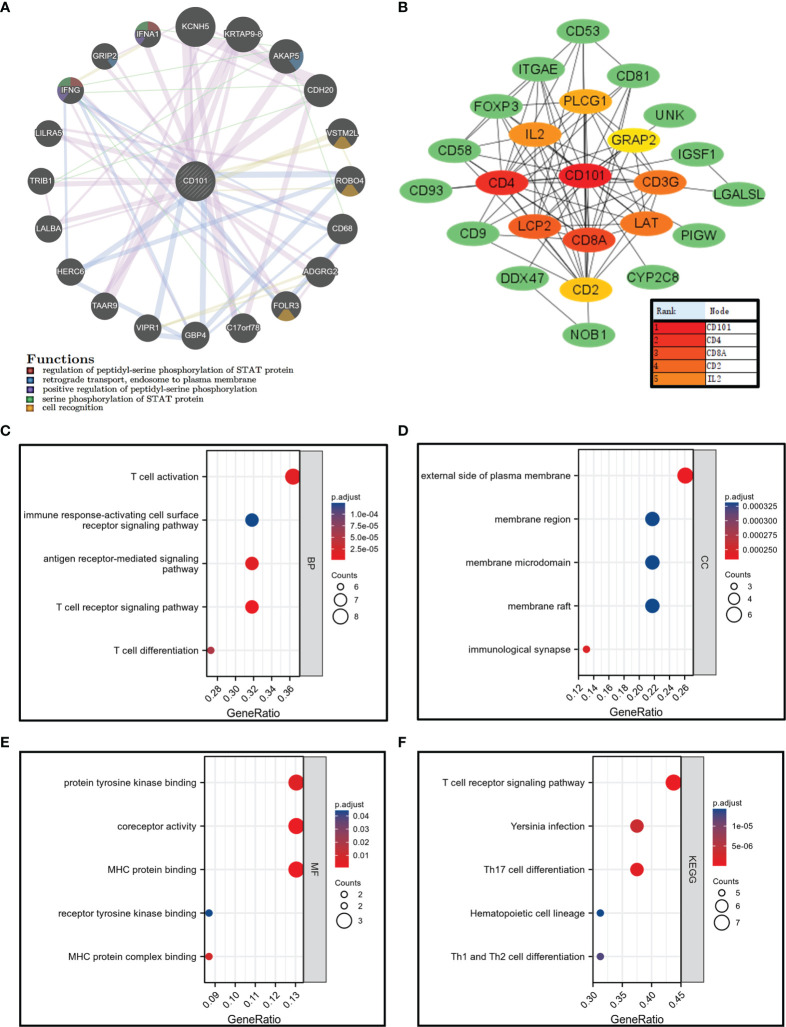
Gene–gene interaction network, PPI network, and enrichment analysis related to binding proteins of CD101. **(A)** CD101 related gene–gene interaction network. **(B)** CD101-associated PPI network. **(C–E)** GO analysis. **(F)** KEGG analysis.

The gene–gene interaction network analysis was performed to identify genes that interacted with CD101 with the highest frequency. The top 20 genes among the list, including KCNH5, KRTAP9-8, AKAP5, and CDH20, were processed to the subsequent enrichment analysis, in which we demonstrated an association of these genes with cell recognition and phosphorylation of STAT protein (**Figure 7A**). Thereafter, the binding proteins of CD101 were also screened using the STRING database and Cytoscape. Correspondingly, additional enrichment analyses with respect to CD101-binding partners were carried out to further explore its biological functions (**Figure 7B**). Consequently, the results indicated that the biological process (BP) included T cell activation, T cell receptor signaling pathway, and T cell differentiation (**Figure 7C**). The cellular component (CC) involved the external side of the plasma membrane, membrane region, and immunological synapse (**Figure 7D**). The molecular function (MF) was mainly enriched in receptor tyrosine kinase binding, MHC protein binding, and MHC protein complex binding (**Figure 7E**). The KEGG pathway analysis revealed pathways in relation to Th1 and Th2 cell differentiation, T cell receptor signaling pathway, and Th17 cell differentiation (**Figure 7F**). Analyses of binding partners of CD101 further strengthen the potential of CD101 in modulating immune responses and ECM formation.

### CD101-Related Immune Cell Infiltration Analysis

**Figure 8:**
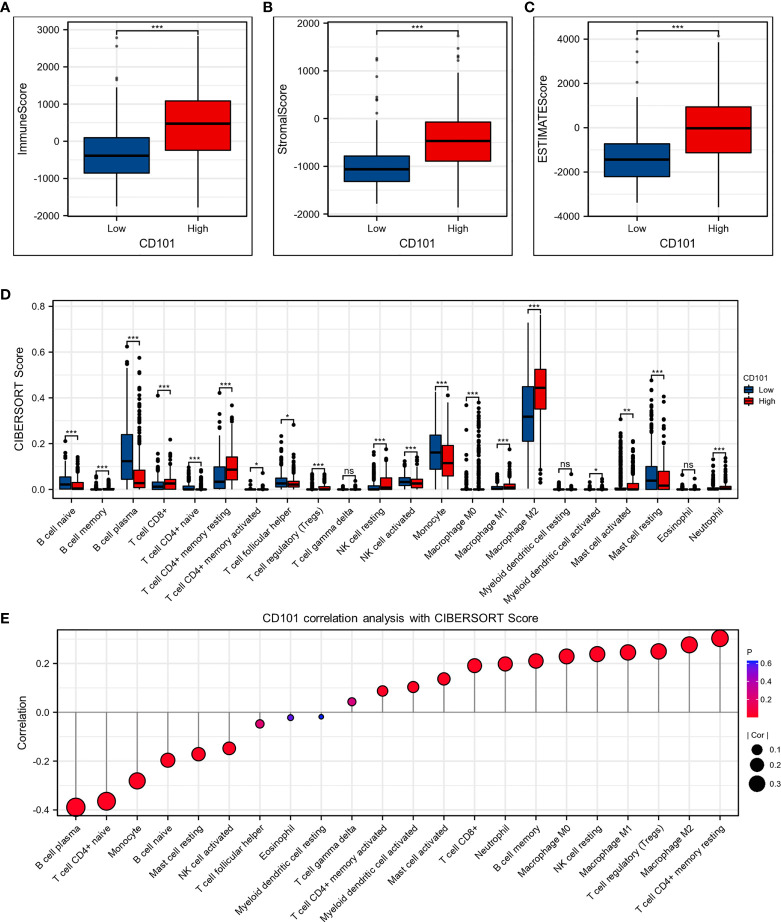
CD101 expression level was associated with unique immune microenvironment in the glioma. **(A–C)** Comparison of ImmuneScore, StromalScore, and EstimateScore between different CD101 expression groups. **(D)** Box plots depicting the CIBERSORT score of 22 immune cells of the high expression group compared to low expression group. **(E)** Correlation analysis between CD101 expression level and CIBERSORT score of 22 immune cells. (ns, *p* ≥ 0.05, *p < 0.05, **p < 0.01, ***p < 0.001).

Since the elevated CD101 expression was demonstrated to correlate with immune alterations and worsening prognosis in glioma patients, we then probed the role of CD101 in remodeling the tumor immune microenvironment. The results revealed that an increased expression of CD101 was associated with significantly higher immune scores (**Figure 8A**), stromal scores (**Figure 8B**), and estimate scores among patients with glioma (**Figure 8C**). To be specific, analysis of putative immune cell infiltration indicated that memory B cells, CD8^+^ T cells, resting memory CD4^+^ T cells, regulatory T cells (Treg), resting (NK) cells, M0 macrophages, M1 macrophages, M2 macrophages, activated myeloid dendritic cells (mDCs), activated mast cells, and neutrophils remained markedly enriched in the high CD101 group (**Figure 8D**). Furthermore, correlation analysis inferring the relationship between CD101 and immune cell infiltration level further validated this point, as evidenced by the potent correlation of CD101 expression with resting memory CD4^+^ T cell, M2 macrophage, Treg, M1 macrophage, resting NK cell, M0 macrophage, memory B cell, neutrophil, CD8^+^ T cell, activated mast cell, activated mDCs, and activated CD4^+^ memory T cell enrichment. Nevertheless, the CD101 expression level was related to the marked decline in activated NK cells, resting mast cells, naïve B cells, monocytes, naïve CD4^+^ T cells, and plasma cells (**Figure 8E**). Taken together, these results implicated that CD101 expression might predict a unique immunosuppressive status of glioma immune infiltration, especially for T cell immune responses.

### Correlation Between CD101 and Immunoregulatory Genes

**Figure 9:**
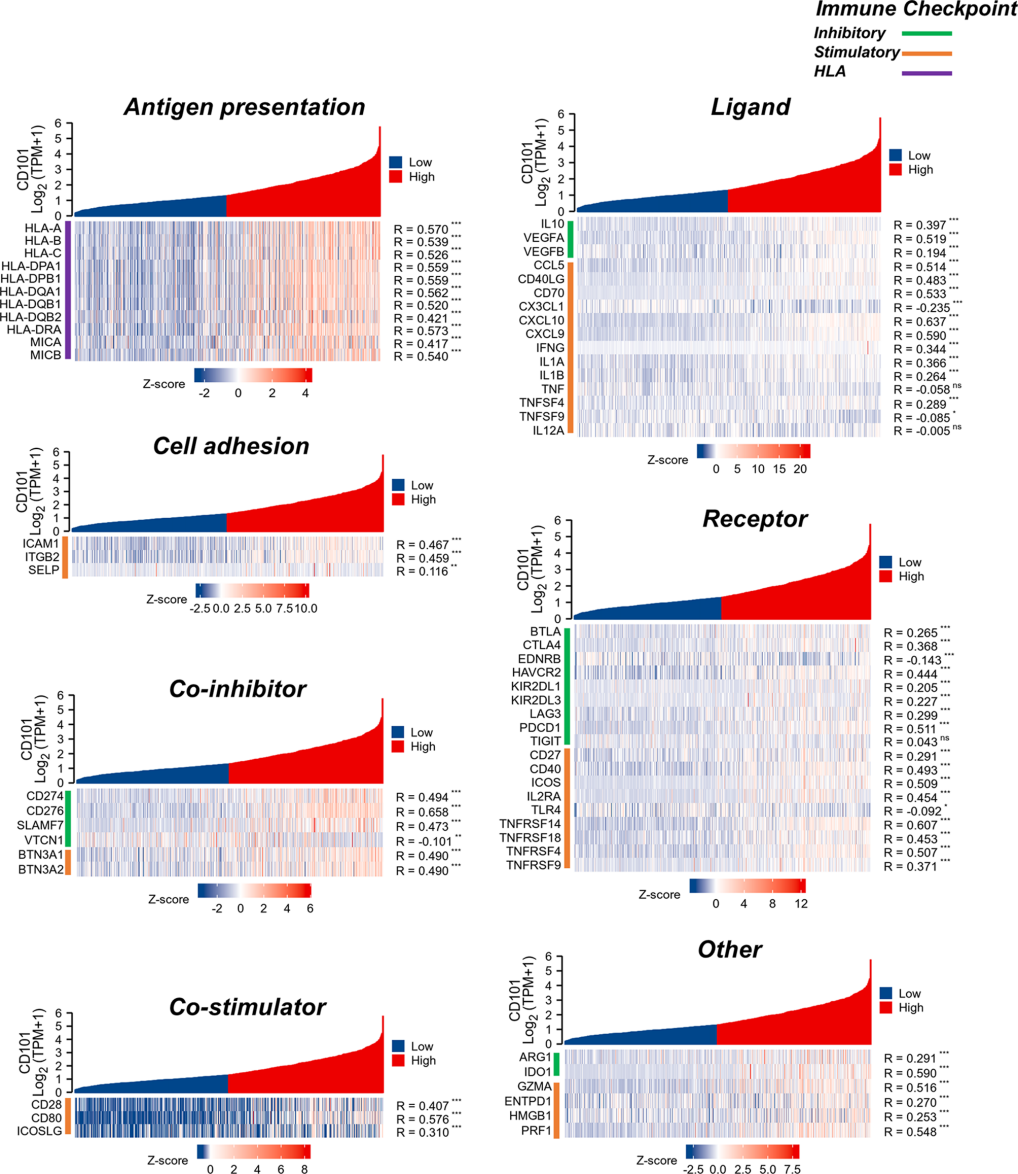
Analysis of correlation between CD101 and immune-related gene in glioma based on the TCGA database. (ns, *p* ≥ 0.05, *p < 0.05, **p < 0.01, ***p < 0.001).

To better understand the immune modulating functions of CD101, we further estimated the correlations between CD101 expression and diverse immunoregulatory molecules in glioma. In line with the study conducted by Thorsson et al. (23), these genes could be categorized into subclasses, including antigen presentation, cell adhesion, co-inhibitory, co-stimulatory, ligand, and receptor. Correspondingly, it showed that CD101 expression could potentially interact with numerous immune-relevant genes, including CD276, CD274, CD80, CTLA4, and PDCD1, implying an immunoregulatory role of CD101 in the glioma immune microenvironment (**Figure 9**).

### Increased CD101 Expression on M2-Like TAMs

**Figure 10:**
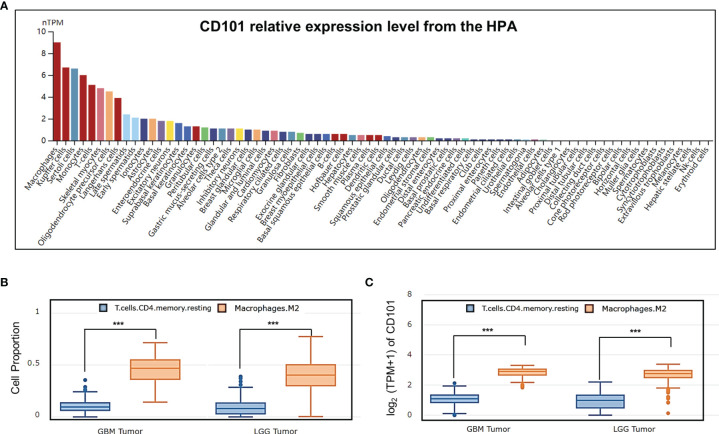
Analysis of CD101 expression based on cell type-level analysis. **(A)** A summary of single-cell RNA normalized expression from all single-cell types in the HPA. **(B)** Cell proportion analysis between M2 and T cell CD4+ memory resting in glioma based on the GEPIA2021 database. **(C)** CD101 expression level analysis between M2 and T cell CD4+ memory resting in glioma based on the GEPIA2021 database (***p < 0.001).

**Figure 11:**
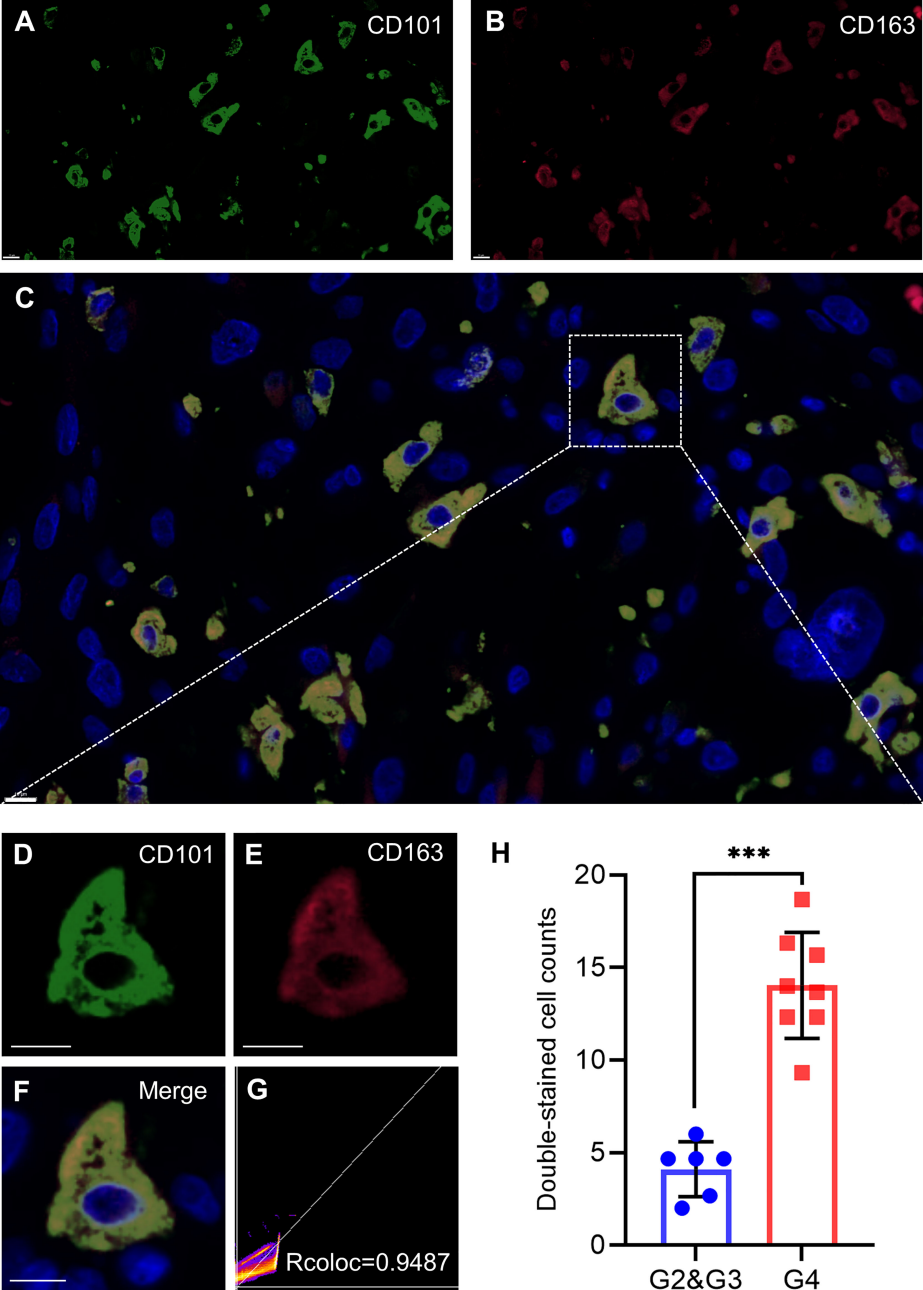
CD101 is a new biomarker on TAMs. **(A–H)** Representative confocal fluorescence microscopy image of CD101 **(A, D)** CD163 **(B, E)**, and merge **(C, F)** in GBM tissue from patients. DAPI (blue) was used for nuclear staining. Scale bar 10 μm. **(G)** Colocalization analysis between CD101 and CD163. **(H)** Double-stained cell counts in glioma with different grades. Statistical significance was determined using the T test. (***p < 0.001).

CD101 expression was manifested to substantially alter immune cell infiltration in glioma TME, prompting us to gain insight into the cellular basis and distribution of CD101. Consequently, single-cell transcriptome-based analysis using the HPA database revealed that macrophages, Kupffer cells, Sertoli cells, monocytes, T cells, skeletal myocytes, and oligodendrocyte precursor cells had a relatively higher expression of CD101 (**Figure 10A**). Based on the results of the correlation analysis inferring immune cell infiltration, the top two cell types, resting memory CD4^+^ T cells and M2 macrophages, were selected for subsequent analysis using the GEPIA2021 database, in which cell type-specific survival analysis confirmed an association of these two types of cells with an unfavorable clinical prognosis (**Supplementary Figures 1A, B**). Meanwhile, analysis of the cellular composition showed that enrichment of M2 macrophages in glioma TME were much more evident than that of the resting memory CD4^+^ T cells, as supported by the result of cell type-specific expression analysis (**Figures 10B, C**). Additionally, we also investigated the difference between Tregs and M2 macrophages, which showed identical results (**Supplementary Figures 2A, B**). Given that, our data strongly implicated that M2-like macrophages in glioma TME were characterized by a high expression of CD101. Correspondingly, *in-situ* immunofluorescence staining was adopted to verify the expression pattern and localization of CD101 in clinical glioma specimens at disparate grades. Immunofluorescence staining of CD163, a well-established marker of M2 macrophage, combined with CD101 demonstrated that CD101 substantially co-localized with CD163, with a Rcoloc of 0.95. More importantly, we further manifested that the number of CD163^+^ CD101^+^ cells was significantly abundant in the grade 4 glioma compared to that in grade 2 and grade 3 gliomas (**Figure 11**).

## Discussion

Glioma was well accepted as the most common type of primary central nervous system (CNS) tumors among adult individuals, accounting for approximately 80% of all malignant CNS tumors (25). Unfortunately, despite tremendous progress made in the diagnosis and managements of glioma, these malignancies typically resulted in a poor quality of life with a dismal clinical prognosis (26). Therefore, it is crucial to decipher the underlying mechanisms that contributed to the malignant phenotype of glioma and to identify robust yet feasible cell-type-specific signatures. In the current study, we confirmed that the CD101 expression was significantly higher in the glioma than that of the normal tissue at both transcriptional and protein levels. Meanwhile, by using Cox regression analysis combined with KM survival analysis, we demonstrated that a high CD101 level served as an independent risk factor in predicting deteriorative OS for glioma patients, as also strengthened by multiple subgroup analyses stratifying patients by WHO grade, integrated diagnosis, age, IDH status, 1p/19q codeletion status, and primary therapy outcome.

To further clarify the functional role of CD101 in glioma, we did an enrichment analysis between high- and low-expression groups in accordance with CD101 mRNA expression. Correspondingly, we identified many terms associated with immune response, including immune effector process, immune receptor activity, regulation of immune effector process, humoral immune response, and primary immunodeficiency. Likewise, results of the GSEA analysis revealed that upregulated CD101 expression was associated with immunoregulatory interactions between a lymphoid and a non-lymphoid cell and interactions between immune cells and microRNAs in the tumor microenvironment. Meanwhile, we manifested that DEGs were also enriched in ECM, regulation of cell adhesion, and ECM–receptor interaction, suggesting that the difference in ECM formations might be observed between two groups. Furthermore, the enrichment analysis implicated that the CD101 level could alter leukocyte migration and chemokine signaling pathway in glioma patients. It has been well-established that the tumor microenvironment in glioma consisted of multiple compartments, including blood vessels, soluble factors, parenchyma cells, infiltrated immune cell populations, and ECM (6, 27). Since the above analysis implied that CD101 expression was associated with immune response and ECM in glioma, it inferred that CD101 is involved in mediating ECM formation and lymphoid-tumor-infiltered myeloid cell interactions, thereby playing a key role in the regulation of immune cell infiltration as well as remodeling of the tumor immune microenvironment of glioma.

In the glioma TME, immune cells are recruited to the neoplastic site and undergo a profound phenotypical shift from an antitumor to pro-tumor state. These pro-tumor immune cells remain abundant in the glioma microenvironment, which are of prominent significance in facilitating malignant growth and therapeutic resistance (28, 29). Herein, we initially reported that a high CD101 expression in glioma is correlated with an increased infiltration of various immune cell types associated with immunosuppression, among which M2 macrophages have attracted our attention. TAMs reportedly played a pivotal role in glioma progression and are identified in high proportions in the landscape of the glioma immune microenvironment. Of note, TAMs were characterized by two major functional subtypes, pro-tumor M2 macrophages and antitumor M1 macrophages, whereas majority of TME-resident TAMs in glioma exhibited M2-like functions (6, 27, 30). Our findings revealed that M2-like TAMs uniquely expressed a high level of CD101, solidifying the relationship between CD101 and immunosuppressive TME in glioma. This point was further strengthened by the bioinformatics analysis using single-cell transcriptome-based data. Moreover, results of immunofluorescence staining showed that CD101 substantially co-localized with CD163. These results implicated that TAMs might manipulate immunosuppressive TME in glioma through upregulating CD101 expression.

Reactivation of the antitumor potential of T lymphocytes represents a well-established therapeutic strategy in treating diverse malignancies (31). In recent years, multiple inhibitors targeting immune checkpoint molecules have achieved remarkable progresses in several cancer types, including PD-1 and CTLA-4 (32). Nevertheless, in a latest phase 3 clinical trial of recurrent GBM, anti-PD-1 therapy failed to exhibit a beneficial effect in comparison with the standard therapy (33). Several factors might contribute to the blunted efficacy of the anti-PD-1 regimen directly or indirectly, including infiltration of immunosuppressive myeloid cells, sequestration of T cells, release of inhibitory metabolites, and glucocorticoid-induced lymphopenia (34–37). These factors reduced T cell effector function commonly referred to as T cell dysfunction or exhaustion (38). Of note, the inhibitory effect of TAMs on T cells has been extensively studied. To be specific, TAMs can express various co-inhibitory molecules that interact with isogenic receptors expressed on T cells, thereby attenuating T cell activation and proliferative capacity. Moreover, TAMs were capable of releasing various inhibitory cytokines that further impaired antitumor functions of T cells (6, 39, 40). Based on a functional network and literature related to CD101 (10, 13, 41), we found that CD101 might have a close relationship with multiple functional markers of T cells, including CD8A, CD4, CD3G, IL2, and FOXP3. Furthermore, enrichment analysis based on CD101 proteins revealed that CD101 is potentially involved in the biological processes related to T cell immune response and antigen presentation. Likewise, gene–gene network analysis implied that *CD101* might regulate the functional status of T cells *via* the cell recognition process. This point was further supported by the putative association of *CD101* expression with phosphorylation of STAT family proteins, which were deemed as critical transcriptional factors determining the activations of many immune cells (39, 42). Based on the above analyses, it demonstrated that TAMs with a high expression level of CD101 might play a pivotal role in inhibiting the antitumor functions of T cells in glioma TME, leading to sustainable immunosuppression.

There are several limitations when we interpreted our findings. Firstly, majority of the analyses were carried out solely using transcriptome-based data. To further clarify the biological role of CD101 in the glioma TME, evidence provided by *in-vitro* functional assays is needed in future studies. Secondly, although we performed a correlation analysis between CD101 expression and immune cell infiltration, there is lack of explanation for the immune infiltration analysis based on different clinical subgroups. Thirdly, we mainly focused on CD101 on TAMs, whereas its expression pattern and functions in other immune cell subsets also deserved in-depth exploration.

Taken together, our results revealed that CD101 could serve as a novel indicator in predicting malignant phenotypes and clinical prognosis for glioma patients. Furthermore, multidimensional bioinformatics analyses and *in-situ* immunofluorescence staining indicated that CD101 was predominantly expressed on M2-like TAMs, in association with remodeling of the glioma immune microenvironment. These results provide insight into the cellular and molecular basis of the glioma immune microenvironment and identify novel therapeutic targets for immune-adjuvant therapies.

## Data Availability Statement

The datasets generated and analyzed in this study can be found in online repositories. The accession number can be found in the article/**Supplementary Material**. Further inquiries can be directed to the corresponding authors.

## Ethics Statement

The studies involving human participants were reviewed and approved by the Institutional Research Ethics Committee of the PLA General Hospital. The patients/participants provided their written informed consent to participate in this study.

## Author Contributions

YYL, RQY, and YS conceived the bioinformatics analysis. YXL, JLL, and HYL were responsible for the data interpretation. YYL, RQY, and LC co-wrote the paper. YMY and YQG undertook the statistical analyses. All authors contributed to the article and approved the submitted version.

## Funding

This work was supported by the Youth Program of the Natural Science Foundation of Hainan Province of China (No. 821QN388); the National Natural Science Foundation of China (No. 81672824, 82172680, 82130062, 81730057 and U20A20380); and the Key Research and Development Program of Liaoning Province (No. 2019JH2/10300036).

## Conflict of Interest

The authors declare that the research was conducted in the absence of any commercial or financial relationships that could be construed as a potential conflict of interest.

## Publisher’s Note

All claims expressed in this article are solely those of the authors and do not necessarily represent those of their affiliated organizations, or those of the publisher, the editors and the reviewers. Any product that may be evaluated in this article, or claim that may be made by its manufacturer, is not guaranteed or endorsed by the publisher.

## Supplementary Material

Supplementary Figure 1 Prognostic value of immune cell infiltration in GEPIA2021 database. **(A)** Survival curves of Macrophage M2 was shown for OS. **(B)** Survival curves of T cell CD4+ memory resting was shown for OS.

Supplementary Figure 2 Analysis of CD101 expression based on cell type groups. **(A)** Cell proportion analysis between M2 and T cell regulatory in glioma based GEPIA2021 database. **(B)** CD101 expression level analysis between M2 and T cell regulatory in glioma based GEPIA2021 database.

Supplementary Table 1 Clinical information of 14 glioma samples.
