# Supplementary figures and images for: Identification of CD101 in Glioma: A Novel Prognostic Indicator Expressed on M2 Macrophages
Source: Front Immunol. 2022 Mar 8;13:845223. doi: 10.3389/fimmu.2022.845223 (PMC8957828; doi:10.3389/fimmu.2022.845223)

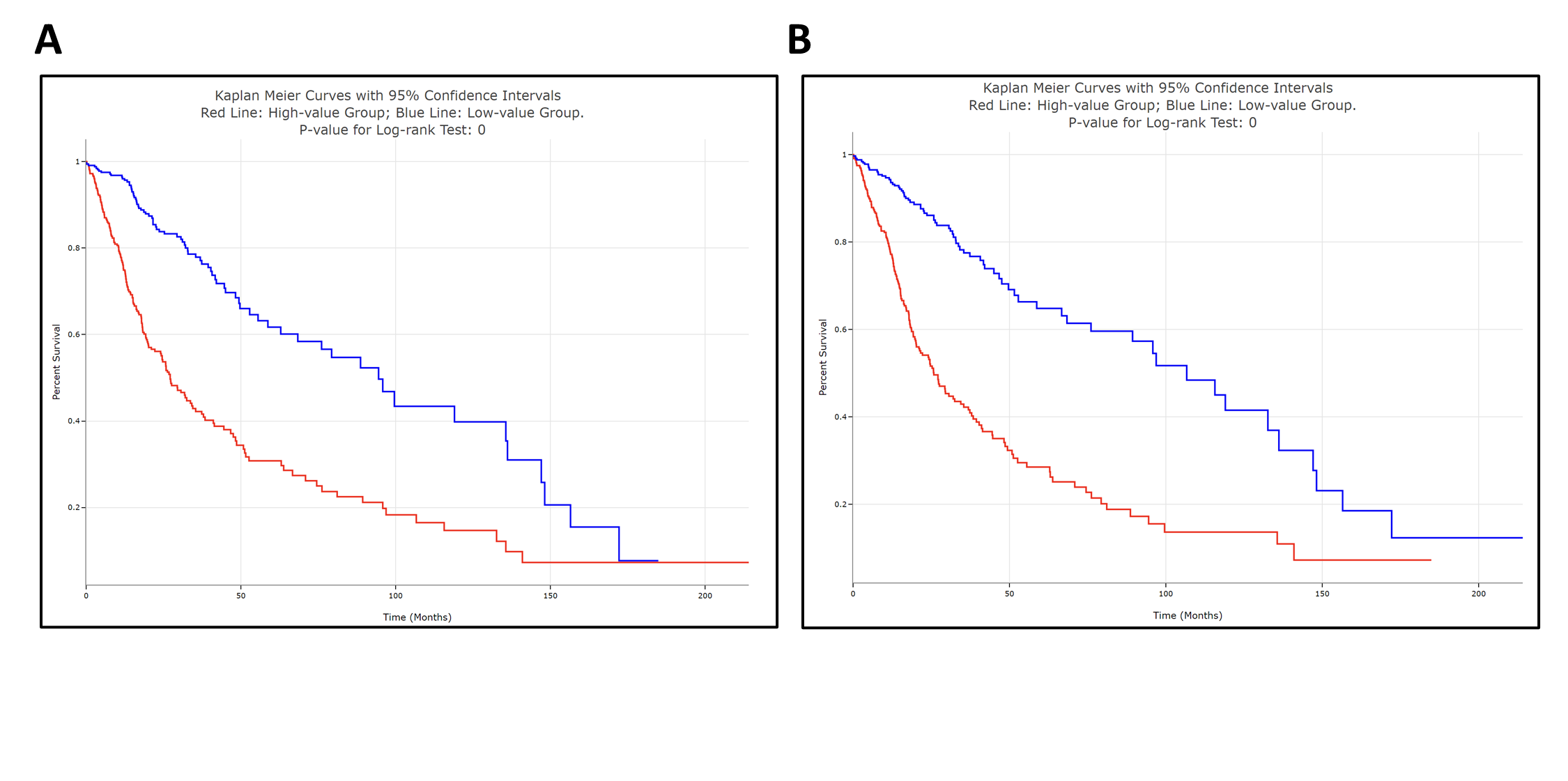

Supplement: Supplementary Figure 1 — Prognostic value of immune cell infiltration in GEPIA2021 database. (A) Survival curves of Macrophage M2 was shown for OS. (B) Survival curves of T cell CD4+ memory resting was shown for OS. [file Image_1.tif]

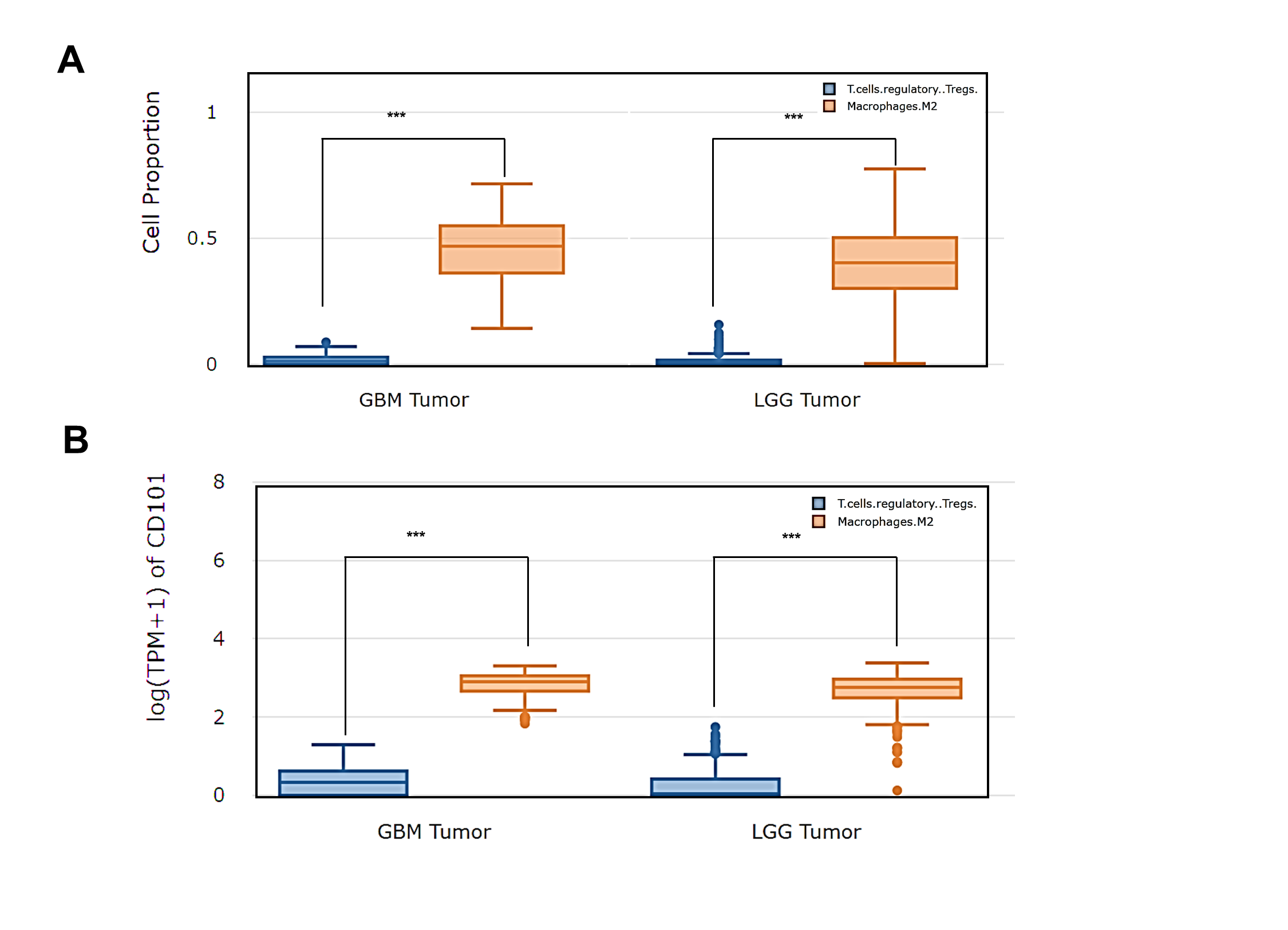

Supplement: Supplementary Figure 2 — Analysis of CD101 expression based on cell type groups. (A) Cell proportion analysis between M2 and T cell regulatory in glioma based GEPIA2021 database. (B) CD101 expression level analysis between M2 and T cell regulatory in glioma based GEPIA2021 database. [file Image_2.tif]
