# Supplementary material for: Identification of CD101 in Glioma: A Novel Prognostic Indicator Expressed on M2 Macrophages
Source: Front Immunol. 2022 Mar 8;13:845223. doi: 10.3389/fimmu.2022.845223 (PMC8957828; doi:10.3389/fimmu.2022.845223)
Supplement: Supplementary Table 1 — Clinical information of 14 glioma samples. [file Table_1.docx]

| **No.** | **Age** | **Gender** | **WHO grade** | **Histological type** |
| --- | --- | --- | --- | --- |
| **1** | 39 | Male | G2 | Astrocytoma |
| **2** | 41 | Male | G2 | Astrocytoma |
| **3** | 53 | Female | G3 | Anaplastic oligodendrocytoma |
| **4** | 39 | Female | G3 | Anaplastic astrocytoma |
| **5** | 40 | Male | G3 | Anaplastic oligodendrocytoma |
| **6** | 43 | Female | G3 | Anaplastic oligodendrocytoma |
| **7** | 56 | Male | G4 | Glioblastoma |
| **8** | 52 | Male | G4 | Glioblastoma |
| **9** | 57 | Female | G4 | Glioblastoma |
| **10** | 44 | Male | G4 | Glioblastoma |
| **11** | 52 | Female | G4 | Glioblastoma |
| **12** | 49 | Male | G4 | Glioblastoma |
| **13** | 33 | Female | G4 | Glioblastoma |
| **14** | 57 | Male | G4 | Glioblastoma |

**Supplementary Table 1| Clinical information of 14 glioma samples**
